# Emotion Access and Navigation in Chinese Couples: Insights and Adaptations From Emotionally Focused Couples Therapy Therapists

**DOI:** 10.1111/jmft.70093

**Published:** 2025-11-21

**Authors:** Chi‐Fang Tseng, Jiayi Liu, Hamed M. Fatahian‐Tehran, Ting Liu

**Affiliations:** ^1^ Human Development and Family Studies Michigan State University East Lansing Michigan USA; ^2^ Philadelphia Center for Emotionally Focused Therapy Philadelphia Pennsylvania USA; ^3^ Asian Association for Emotionally Focused Couple and Family Therapy Taiwan

**Keywords:** Chinese couples, cultural adaptation, emotionally focused couple therapy, therapists' perspectives

## Abstract

The effectiveness of Emotionally Focused Couple Therapy (EFCT) has been well‐documented, yet its application with Chinese couples remains underexplored. This is the first qualitative study examining therapists' perspectives on using EFCT with Chinese couples in clinical settings. Thematic analysis of semi‐structured interviews with 20 therapists from mainland China, Taiwan, and Hong Kong revealed that therapists viewed EFCT as a useful approach for Chinese couples. EFCT was reported to be helpful for couples with relationship distress and mental health concerns. However, therapists emphasized the importance of understanding cultural values and nuances that influence presenting problems. Overall, therapists recommended adjusting EFCT skills to better serve the needs of Chinese couples, understanding cultural impacts on couples' presenting problems, and incorporating additional components and training for therapists working with Chinese couples. These findings provide a foundation for culturally adapted EFCT, ensuring its relevance for Chinese couples while honoring their unique relational and cultural contexts.

## 1 Introduction

Emotionally focused couple therapy (EFCT; Johnson 2019) is an evidence‐based intervention that has been shown to be effective for various mental health conditions, including depression (e.g., Alder et al. 2018; Wittenborn et al. 2019) and PTSD (e.g., Ganz et al. 2022; Weissman et al. 2017). EFCT has also proven effective in addressing relationship distress (e.g., Dalgleish et al. 2015), with a meta‐analysis demonstrating medium to large treatment effects from pretest to posttest and from posttest to follow‐up for couples experiencing relationship distress (Spengler et al. 2024). Although EFCT outcome research has primarily been conducted in North America, its application is gradually expanding to other regions, including Asia. To date, there is only a few studies involving clinical trials to examine the effectiveness of EFCT in Asian contexts, including in Taiwan (Tseng et al. 2024) and Iran (e.g., Soltani et al. 2013; Soleimani et al. 2015).

In a recent pragmatic clinical trial of EFCT with couples in Taiwan, Tseng et al. (2024) found that while depressive symptoms decreased over the course of treatment, relationship distress did not show a significant reduction—the finding was inconsistent with research conducted in North America, which has consistently shown that EFCT improve relationship distress. Further investigation into the predictors of change (i.e., traditionalism, attachment, and emotional expressivity) in relationship distress (Tseng et al. 2025) revealed that couples who exhibited high emotional expressivity at intake experienced a reduction in relationship distress over time, and women who adhered to more traditional gender roles similarly saw a decrease in their relationship distress. These findings suggest that certain client characteristics, especially the level of emotional expressivity and gender roles, may influence EFCT outcomes when applied in Asian cultural contexts. However, our understanding of how these and other cultural factors affect treatment outcomes remains limited.

Over the past decade, a growing number of therapists in East Asia have been trained in EFCT and have successfully used it in their work with couples. Specifically, more than 2,100 therapists have been trained in mainland China, 1,400 in Taiwan, and 700 in Hong Kong (T. Liu, Director of the Asian Association for Emotionally Focused Couple and Family Therapy, personal communication, February 12, 2025). Despite this growth, more existing research on EFCT with Chinese couples has primarily been limited to case studies (e.g., Liu and Hung 2019), process research on therapists' use of EFCT skills (e.g., Hung 2022), and couples' experiences of receiving EFCT (e.g., Sun 2019), rather than outcome research.

Although the number of EFCT‐trained therapists working with Chinese couples continues to grow, empirical evidence supporting EFCT's effectiveness in this cultural context remains limited, with only two outcome studies to date (Tseng et al. 2024, 2025), and Tseng et al. (2024) already showed findings inconsistent with those from North America. This makes our current study important as we need to better understand therapists' perspectives on how EFCT works, what challenges arise when applying EFCT, how Chinese cultural nuances might shape the therapy process, and how EFCT might be adapted to better fit with Chinese culture. As Wiltsey Stirman et al. (2019) emphasized, practitioner input is a crucial first step in the cultural adaptation of interventions. These insights will serve as a foundation for culturally adapting EFCT to better meet the unique needs of Chinese couples.

## 2 Chinese Contexts and EFCT

It is important to critically examine how EFCT translates into the Chinese cultural context, as Western and Chinese cultures differ significantly in many dimensions, particularly in their orientations toward individualism and collectivism (Dion and Dion 1993). These cultural orientations shape not only individual values but also the dynamics and expectations of intimate relationships.

In individualistic cultures, such as those in many Western societies, intimate relationships often prioritize personal goals, open emotional expression, and autonomy (Dion and Dion 1993; Hamamura et al. 2018). Partners are generally expected to focus on each other, placing less emphasis on extended family or broader social obligations and expectations (Dion and Dion 1993; Hamamura et al. 2018). However, collectivistic cultures, such as Chinese society, tend to view intimate relationships as embedded within larger family system, especially including extended family as part of the family system (Du et al. 2023; Hu and Peng 2015). In these contexts, maintaining relational harmony, fulfilling social roles, and meeting family and societal expectations are often prioritized, even when doing so requires individuals to set aside their personal needs or tolerate their partners or unsatisfying relationships (Chen and Li 2007; Dion and Dion 1993). In addition, research has also shown that Chinese couples are more likely to engage in indirect communication and emotional suppression, whereas the direct and emotionally expressive style are often encouraged and observed in Western societies (Halford et al. 2018; Tsai et al. 2006).

EFCT emphasizes open expression of vulnerable emotions and individual attachment needs, and change in EFCT occurs when couples experience new, corrective emotional experiences (Johnson 2019). Because emotional expression plays a central role in EFCT, and given that the model was originally developed within an individualistic cultural framework, it is important to examine how its core principles and interventions align with the needs of couples in collectivist contexts—particularly through insights from therapists who have firsthand experience applying EFCT with Chinese couples. Without such cultural understanding from therapists, there is a risk that EFCT may be applied in ways that do not fully align with the relational expectations and values of Chinese couples.

## 3 Emotion Access and Navigation in EFCT

EFCT employs specific therapeutic interventions, including reflection, validation, evocative responding, and empathic conjecture, to help clients engage deeply with their emotional experiences and create meaningful changes (Johnson 2019). Reflection is a fundamental skill in EFCT, where the therapist mirrors the client's emotional experiences, offering back what the client expresses in a clearer, more focused manner (Johnson 2019). This process enables clients to name and understand their emotions, thus enhancing their emotional awareness (Greenberg and Johnson 1988). Another EFCT skill, validation, plays a crucial role in creating a safe therapeutic space, where clients feel that their emotional experiences are legitimate and meaningful. When therapists validate clients' emotions, they communicate acceptance and understanding, reducing feelings of shame or defensiveness (Greenberg and Paivio 1997).

Two advanced EFCT skills are also helpful in facilitating emotion access and navigation in therapy. Evocative responding, designed to elicit deeper emotional responses from clients. Through carefully phrased questions or observations, the therapist helps clients access emotions that may lie beneath the surface of their awareness (Greenberg and Johnson 1988). Empathic conjecture is another key tool to allow therapists to offer tentative hypotheses about the client's emotional experience, often suggesting emotions that clients may not yet have fully articulated or understood (Greenberg and Johnson 1988). Empathic conjecture is particularly effective in moments where clients may feel emotionally stuck, as it encourages them to consider new emotional perspectives and meanings (Greenberg and Paivio 1997; Johnson 2019).

These EFCT techniques—reflection, validation, evocative responding, and empathic conjecture—form a comprehensive approach to emotional access and navigation. However, how these techniques work and fit when applied to Chinese couples remains not fully understood. Therapists' input on how they perceive these techniques as helpful or in need of adaptation can offer valuable insights into the culturally attuned application of EFCT skills with Chinese couples.

## 4 Current Study

The current study aims to expand the existing literature by examining therapists' perspectives on applying EFCT with Chinese couples—identifying what works, what fits, and what does not. We first provide an overview of the common presenting problems among Chinese couples in clinical settings, offering context on the population therapists serve. We then focus on therapists' perspectives regarding the application of EFCT with Chinese couples. Our two main research questions are:

1. What are the most common presenting problems among Chinese couples in clinical settings in Asia?
2. What aspects of EFCT work or do not work with Chinese couples, and what cultural adaptations do therapists recommend?

## 5 Method

We employed a constructivist perspective in our qualitative research design, which emphasizes the values of individuals' interpretive processes and views knowledge as co‐created through the interaction between researchers and participants (Lee 2012). This approach acknowledges that therapists' perspectives are constructed through their interactions with couples, their training, and the larger social contexts, allowing us to build a contextually grounded understanding. We followed the Reporting Standards for Qualitative Research (JARS‐Qual; Levitt et al. 2018) in reporting the results. This study was approved by the Michigan State University institutional review board (study # 00009966).

### 5.1 Participants

Twenty EFCT therapists from Taiwan (*n* = 8, 40%), mainland China (*n* = 8, 40%), and Hong Kong (*n* = 4, 20%) were included in the current study. Table 1 shows the demographic information of each participant. The average age of therapists is 44.4 years (SD = 5.5, range = 36–60). There are fifteen (75%) female and 5 (25%) male therapists. Two (10%) identified as bisexual, one (5%) identified as lesbian, and the rest (85%) identified as heterosexual. Sixty percent of therapists reported being married. Twenty percent obtained a Bachelor's degree, 65% obtained a Master's degree, and 15% obtained a doctoral degree. Participants' type of licensure varies by regions. All the Taiwanese therapists have a licensure in Counseling Psychologist. Among the 12 therapists in mainland China and Hong Kong, eight are national level 2 Psychological Counselors, three are Registered Social Workers, and one does not have a licensure (In Hong Kong, the law does not require one to obtain mental health licensure to practice mental health treatment). All the therapists are EFCT certified therapists, and 55% are also EFCT certified supervisors. At the point of being interviewed, therapists had seen clients for 15.0 years (SD = 5.3, range = 8–29) and used EFCT for 10.5 years (SD = 3.2, range = 5–16) on average. The sample consists primarily of therapists practicing in private practice settings (95%). Some therapists engage in additional contexts, such as agencies (10%), college counseling centers (10%), government social welfare departments (5%), and referrals from courts (5%). The hourly therapy fees therapists charged from couples ranged from USD $0 to $169, with an average of $106.0 (SD = $43.7). The number of EFCT sessions reported by therapists varied considerably across cases. The mean number of sessions ranged from 11.3 to 20.4 (SD = 5.1–7.5).

**Table 1:** Demographic information of the EFCT therapists.

ID	Region	Age	Gender	Sexual identity	Marital status	Education level	Licensure type	EFCT certification	Years seeing clients post EFCT training	Years seeing clients	Work settings	Therapy fee (USD/hr; approx. equivalent)
1	TW	41	W	HT	M	Master	CP	CT + CS	13	15	PP	82
2	TW	42	W	HT	M	PhD	CP	CT + CS	12	16	PP	117
3	TW	44	W	HT	NM	Master	CP	CT + CS	14	19	PP	83
4	TW	44	W	HT	NM	PhD	CP	CT + CS	15	22	PP + GR	90
5	TW	47	W	HT	M	Master	CP	CT + CS	16	17	PP	169
6	TW	45	W	BI	NM	Master	CP	CT + CS	10	22	PP + AG + CCC	28‐82
7	TW	36	W	LES	NM	Master	CP	CT + CS	11	11	PP + CCC	25‐82
8	TW	49	W	HT	M	Master	CP	CT	7	20	PP + GR	94
9	CN	43	M	HT	M	Bachelor	NL2PC	CT	11	15	PP	169
10	CN	41	W	HT	M	Master	NL2PC	CT + CS	9	11	PP	113
11	CN	41	M	HT	M	Master	NL2PC	CT	7	11	PP	169
12	CN	44	W	HT	NM	Bachelor	NL2PC	CT	8	12	PP	113
13	CN	36	W	BI	NM	Bachelor	NL2PC	CT	13	8	PP	113
14	CN	46	W	HT	M	Bachelor	NL2PC	CT	12	13	PP	113‐169
15	CN	38	M	HT	M	Master	ISW	CT	5	10	PP	85
16	CN	45	W	HT	NM	PhD	NL2PC	CT + CS	11	15	PP	141
17	HK	45	M	HT	M	Master	NL2PC	CT + CS	8	10	PP	94‐103
18	HK	60	M	HT	M	Master	NA	CT + CS	13	13	PP	154
19	HK	50	W	HT	M	Master	RSW	CT	5	29	PP	42‐113
20	HK	51	W	HT	NM	Master	RSW	CT	9	10	AG	0

Abbreviations: AG = agency, BI = bisexual, CCC = college counseling center, CN = mainland China, CP = counseling psychologist, CS = certified supervisor, CT = certified therapist, GR = government referral, HK = Hong Kong, HT = heterosexual, ISW = intermediate social worker, LES = lesbian, M = man, M = married, NL2PC = National Level‐2 Psychological Counselor, NM = not married, PP = private practice, RSW = Registered Social Worker, TW = Taiwan, W = woman.

### 5.2 Procedures

#### 5.2.1 Research Team

We valued research reflexivity in both data collection and analysis processes. We recognize that our interpretation of participants' experiences is constructed through both our interactions with participants and our own cultural and training background. Given our background, we acknowledge that bias in data collection and data interpretation might exist even with our intentional effort to reduce it. We provide our positionality and background below.

The first two authors are the main contributors to the data analysis and interpretation. The first author is a faculty member at a US institution, holding both master's and PhD degrees in couple and family therapy. She is a licensed counseling psychologist in Taiwan and a licensed marriage and family therapist in the United States, with clinical and research expertise in EFCT and qualitative research. Bilingual in Chinese and English, her experiences conducting therapy in both Taiwan and the United States, along with her research on the effectiveness of EFCT with couples in Taiwan, have motivated her to examine the suitability of EFCT for non‐US populations. The second author is a PhD candidate in the field of human development and family studies as well as a Chinese national with professional training in both mainland China and the United States. Through her studies in the United States, she gradually became aware of cross‐cultural differences in family dynamics between Chinese and European American's families. This experience sparked her research interest in exploring how families function across cultures and how interventions can be adapted to meet the needs of Chinese families. The third author is a certified EFCT therapist with many years of experience practicing EFCT. He has been delivering EFCT for Asian couples and training therapists in EFCT in Iran. The fourth author is a certified EFCT therapist, supervisor, and trainer, as well as the director of the Asian Association for Emotionally Focused Couple and Family Therapy. She offers EFCT training, supervision, and case consultation to therapists in mainland China, Hong Kong, Taiwan and Singapore in Mandarin. She specializes in the multicultural application of EFCT.

We acknowledge that our professional investment in EFCT and EFCT community in Asia may influence the way we interpret participants' perspectives or overlook instances where EFCT may have been less effective. To mitigate this, we engaged in ongoing reflexive dialogs throughout the research process and consistently challenge our assumptions and interpretations.

#### 5.2.2 Data Collection

Participant recruitment was conducted in collaboration with the fourth author, the director of the Asian Association for Emotionally Focused Couple and Family Therapy, who assisted in identifying three EFCT certified therapists in mainland China, Taiwan, and Hong Kong to serve as research liaisons. We chose these three regions because our research aimed to examine EFCT certified therapists who use EFCT with Chinese couples, and these regions currently have established EFCT communities with certified therapists and supervisors who predominately work with Chinese couples (Asia EFT 2025). The research team then reached out to these liaisons, set up meetings to discuss the research purposes and procedures, and obtained their consent to help identify other eligible EFCT certified therapists or supervisors for this interview study. Eligible participants were required to be at least 18 years of age, provide couple therapy for Chinese couples, have completed an EFCT Externship and Core Skills training, be certified as EFCT therapists, and use EFCT as their primary clinical intervention for couple therapy.

Each research liaison from each region received $100 as compensation for their time and effort in supporting the recruitment process. The liaisons used purposeful sampling to provide a list of 6‐8 eligible EFCT therapists and supervisors who might be interested in participating. The first author then emailed each potential participant with the research information and consent form, with all materials provided in Chinese. Eligible participants were invited to join a 1‐ to 1.5‐h Zoom interview and received a $100 gift card upon completing the interview. Once therapists consented to participate, they were officially enrolled in the study. The first author then conducted all the interview with participants.

#### 5.2.3 Data Analysis

We employed a thematic analysis approach to identify patterns within the data (Braun and Clarke 2022). Built on previous research about application of EFCT among Chinese couples, we incorporated both deductive (i.e., theory‐driven) and inductive (i.e., data‐driven) approaches to generate codes. The deductive approach guided the development of initial interview topics. As previous studies suggested the effectiveness of EFCT in certain clinical outcomes (e.g., depression, relationship satisfaction) among couples (Spengler et al. 2024), we specifically explored therapists' experiences in these domains to assess whether their experiences aligned with the literature. Additionally, informed by cross‐cultural literature highlighting reserved emotion expression in Chinese cultures (e.g., Kraus et al. 2024), we designed interview questions exploring therapists' experiences using certain techniques that involve emotion expression. To gain a comprehensive understanding of therapists' experiences, on the other hand, we also included open‐ended questions in the interviews, allowing additional codes (e.g., other clinical outcomes, application of other EFCT techniques, the impact of cultural values on presenting problems) to emerge organically from the data. Codes were mainly constructed at the semantic level to align closely with therapists' perspectives. We used ATLAS.ti 24.0 (ATLAS.ti Scientific Software Development GmbH 2024) qualitative coding software to assist in the coding process.

We followed the six phases suggested by Braun and Clarke (2022), with an iterative process. The first author conducted all interviews, and the second author watched all video recordings and proofread the transcripts to ensure prolonged engagement with the data. Both the first and second authors created memos reflecting their thoughts during this phase. Then, five transcripts (two from Taiwan, two from mainland China, and onr from Hong Kong) were selected to generate initial codes. The first two authors independently developed codes for all research questions and then discussed their codes to reach a consensus. During this phase, a codebook was developed, organized into three sections: (1) Chinese couples' presenting problems, (2) EFCT in improving clinical outcomes, and (3) recommendations for cultural adaptation of EFCT. The first and second author applied this codebook to the remaining transcripts, meeting regularly to discuss interpretations of direct quotes and potential themes. Themes were developed and reviewed to ensure they accurately and coherently represented the data. After discussion, sub‐themes were added. All coding was conducted in the participants' original language. In the final phase, the first author selected representative quotes for each theme and translated them into English. The second author proofread the translations to ensure accuracy in conveying the cultural meanings embedded in the quotes.

#### 5.2.4 Rigor

We implemented several strategies to ensure the rigor of our findings (Nowell et al. 2017). First, we intentionally selected participants from different Chinese‐speaking regions in Asia and reported demographic variations in therapists' experiences to enhance the representativeness. Second, we used researcher triangulation, involving constant discussion of diverse perspectives between multiple researchers, to strengthen the credibility of the themes identified in the collaborative data analysis process. Third, we emphasized researcher reflexivity throughout the study process. We used memos and discussions to reflect on how our upbringing and understanding of Chinese couples might affect our interpretation of participants' narratives.

## 6 Results

In this result section, we will address two research questions by organizing this section into two parts: (1) the most common presenting problems among Chinese couples, and (2) therapists' perspectives on what aspects of EFCT work well with Chinese couples, what challenges they encounter in using EFCT, and their recommendations for cultural adaptations.

### 6.1 The Most Common Presenting Problems Among Chinese Couples

#### 6.1.1 Infidelity

Sixteen out of twenty therapists reported that infidelity is the most common presenting problem they observe in their clinical practices. Infidelity is not limited to sexual and intimate relationships with an extramarital partner; it also includes male partners paying for sex work. One cultural factor adding complexity to this issue is that, in some regions, seeking sex services is considered part of the business routine. As one therapist in mainland China shared, “For men, seeking sex services is a way to network in their work” (P11, CN).

Therapists also discussed that most couples had been aware of infidelity for a long time, but their coping strategy was often tolerance. By the time they seek therapy, it is often too late to repair the relationship, as they have already endured significant pain (we will discuss the strategy of using tolerance to maintain relationships among Chinese couples later in the result section):

So usually, one partner is often very eager [to start therapy] because their tolerance for the infidelity has already reached its limit. As a result, the other partner, even if they don't believe in the [therapy] process, ends up being invited by their partner to come. Sometimes, this invitation can even feel like a threat—if they don't come to therapy, the consequence might be divorce.

(P2, TW)

#### 6.1.2 In‐Laws' Involvement in Couple's Relationships

Another common presenting problem noted by therapists is issues related to in‐laws. These challenges often involve both partners' families of origin, with a particular emphasis on conflicts between the female partner and her mother‐in‐law. Later in the result section, we will share more nuances of this phenomenon. Therapists shared that they had couples come to therapy complaining about the mother‐in‐law trying to interfere in the couple's relationship, directing her son not to do certain things or not to treat his wife in a certain way. A therapist from mainland China shared:

In some cases, (the mother‐in‐law) attempted to break up the couple. And there are also cases involving unmarried couples, where parents disapprove of the relationship…Then there are situations where one party isn't ready to get married, but due to parental pressure, they feel forced to get married. There's also pressure from family elders or relatives, urging the couple to get married, have children, or start a family…… These often lead to significant conflicts in their relationship.

(P19, CN)

#### 6.1.3 Parenting

Parenting is another common presenting problem that leads couples to seek therapy. These conflicts often include decisions about whether to have a child, disagreements on parenting approaches once a child is born, and struggles with teamwork when children face behavioral or mental health issues. One therapist from mainland China noted: “A big presenting problem couples come to therapy for is parenting…either their children are having some behavioral or mental health problems, like school refusal, and then couples don't have agreement or consensus on parenting which leads to high conflict” (P18, CN).

Similarly, a therapist in Taiwan observed that the arrival of a new child often causes significant strain on couples, bringing unresolved issues to the surface: “Because the new baby arriving, couples' relationship satisfaction starts to decrease, or decrease in their intimacy, and having a lot of argument. Some also reported financial challenges because of having a new child, and they started to have disagreement in finance in the family”.

(P1, TW)

#### 6.1.4 Couples' High Conflict

Therapists reported that many couples seek therapy due to high levels of conflict. These conflicts often begin during the dating phase, with couples questioning whether they still want to get married, or during marriage, with conflicts escalating to the point where they contemplate divorce. Such conflicts typically persist for a long time before couples decide to pursue therapy: “Long‐term and high‐level conflict [are very common], leading to one partner wants to leave but one partners wants to stay, and then they'll come to therapy” (P6, TW). For some couples, therapy serves as a means to navigate the logistics of separation or divorce. A therapist from Taiwan noted:

While one or both partners may not want a divorce, it's not because of emotional attachment but rather due to practical reasons, such as shared assets or children—especially assets. For instance, if a couple has shared property or a business, divorce becomes more complex because of asset distribution…. In these scenarios, I make it clear to them about what I can offer as a therapist and what cannot be achieved through therapy…. and my initial sessions are about clarifying the goals of therapy.

(P8, TW)

#### 6.1.5 Trauma

Therapists also reported trauma as a common presenting problem among couples seeking therapy. Many couples come to therapy with a history of trauma, which often stems from their family of origin or childhood experiences. During therapy, couples often come to the realization the impact of trauma on their presenting problems. One therapist from Taiwan noted:

I think there's another unique aspect—for example, when a partner's childhood trauma comes into play in terms of their presenting problems. Simply put, their challenges in the intimate relationship stem from past experiences, such as their family of origin or earlier traumatic events. For instance, they may have childhood trauma that has led to depression.

(P3, TW)

#### 6.1.6 Sex Issues

Sex issues were also mentioned by therapists as one of the common presenting problems. When cultural factors are taken into account, these issues often become even more complex and challenging to address in therapy. Therapists shared that in Chinese culture, open conversations about sex are often considered a taboo, which can lead to a lack of sexual education, limited sexual communication between partners, and shame or avoidance around sexual dissatisfaction. Additionally, cultural expectations around parenting—such as mothers prioritizing the child over the couple relationship—can further strain sexual intimacy. A therapist from Hong Kong noted:

I find that in Hong Kong because the area of living is so tight, so usually after the female partners gave birth to a baby, she will sleep with the child but not the husband so the couple seldom sleep together. Couples live in separate rooms. So recently I find it's so common that the marriage will be like they didn't have sex anymore after the female partner gave birth to a child… I recently have a couple, the wife sleeps in the same room with her 14 years old son.

(P25, HK)

#### 6.1.7 Depression and Anxiety

Therapists shared that couples sometimes present with relationship issues compounded by depression or anxiety. A few therapists noted that some partners were already taking psychotropic medication for their symptoms before beginning therapy. One therapist shared that EFCT not only helps with their relationship issues but also further helps to decrease depressive symptoms: “One partner of the couple had depression for 30 years, but after a couple of sessions 1 day the wife told me that her symptoms has improved and her psychiatrist has decided to decrease the dose of her antidepressant” (P4, TW).

Therapists also noted that anxiety is a common co‐occurring issue alongside relationship distress. Several therapists observed that the combination of medication and EFCT seems to help reduce anxiety symptoms and improve overall relational dynamics:

I think anxiety is something we commonly encounter in therapy…their level of anxiety is typically not low—often moderate—and they may already be taking anti‐anxiety medications, which is quite common. Couples therapy can be quite effective in addressing anxiety. It provides a space for these individuals to process their anxiety within the relational dynamic, often helping both partners understand how the anxious partner's behaviors and emotional needs are influencing the relationship.

(P10, CN)

### 6.2 What Works, What Challenges Arise, and Recommendations for Culturally Adapting EFCT

Overall, EFCT therapists in Asia reported that couples experience significant improvements through EFCT. However, therapists also reported challenges when applying EFCT, often due to cultural differences in values and characteristics that led to challenges in using EFCT, leading to therapists adjusting some of their EFCT skills to fit the needs of couples. Therapists also shared the importance of having a better understanding of cultural values and beliefs of couples are keys to success in EFCT delivery and provided some recommendations and adaptation for therapists who would work with Chinese couples. Lastly, therapists also shared their insight regarding what could be added to the current EFCT model or training to better serve the needs of Chinese couples. In this section, we'll first discuss what aspects of EFCT work well with Chinese couples. We'll then discuss the challenges along with the adaptations in EFCT.

#### 6.2.1 Using EFCT as a Helpful Tool for Working with Chinese Couples

##### 6.2.1.1 Reframing Extended Family as Stressors and Strengthening Partner Alliance

EFCT therapists commonly noted that EFCT techniques can be beneficial for Chinese couples grappling with challenges tied to traditional familism values. EFCT therapists first encourages Chinese couples to shift their focus from external family relationships to the dynamics of their own relationship. Through conceptualizing familism‐related problems as culturally unique stressors, EFCT therapists observed that issues such as conflicts between mothers‐ and daughters‐in‐law or divergent parenting styles often reflect underlying relational dysfunction between spouses. As therapist from mainland China explained:

We often say that mother‐in‐law and daughter‐in‐law conflicts are always a couple's issue. Because no matter how the mother‐in‐law behaves, the key is whether the husband and wife are united. Even if the mother‐in‐law is difficult and interferes in their marriage, if the husband understands his wife and the wife also empathizes with her husband's struggles, they can still have a good marriage.

(P11, CN)

When therapists guided couples to recognize that these conflicts pointed to deeper relational issues between them, couples would focus on improving their emotional bond and reaching a shared understanding, thereby strengthening their ability to manage external pressures. Therapist P18 from mainland China noted that: “in the end, what partners are really fighting over is: Do you have a space for me in your heart?” EFCT therapists help couples recognize their partner's needs, reaffirm their significance, and strengthen their bonds.

##### 6.2.1.2 Recognizing and Validating Unmet Needs in Self and Partner

EFCT also helps Chinese couples recognize each other's emotional needs while accessing and discussing their own. Therapists observed that, due to the cultural tendency to dismiss negative emotional experiences and suppress emotional expression, EFCT's emphasis on emotion is particularly beneficial for Chinese couples whose emotional needs have long gone unseen and unmet. Several therapists highlighted that explicitly explaining the rationale for focusing on clients' emotions helps couples begin to appreciate the role of emotions in their relationship and the problems they face. EFCT thus helps individuals who are unaccustomed to reflecting on their deep feelings, gradually experience, and later articulate, their vulnerable emotions. For instance, therapist P12 from mainland China shared: “For male partners, as they gradually figure out their inner confusion, even though they still find it difficult to express their emotions, they tear up.”

Demonstrating vulnerability is often a precursor to openly expressing it, which then creates a space for partners to better understand and connect with their partners. In response, partners often soften their attitudes and express their care in culturally appropriate ways. Once partners feel that their painful emotions are acknowledged, supported, and validated, they gradually feel safe and more comfortable sharing their vulnerabilities within the relationship. In this way, EFCT disrupts the negative cycle in which couples fail to recognize each other's suffering, feel unheard, and ultimately experience helplessness and emotional insecurity.

#### 6.2.2 Adjusting EFCT Skills to Better Serve the Needs of Chinese Couples

##### 6.2.2.1 Prioritize Validating Cognition to Foster Emotion Access and Engagement

Many therapists reported challenges in helping partners directly express their emotions during sessions—an essential component of EFCT, which aims to facilitate emotional access and sharing. For some partners, storytelling or focusing on cognition often occurs when therapists inquire about their emotions. As one therapist from mainland China noted: “When it comes to men expressing emotions, they usually share an opinion or talk about an event, mostly staying on the cognitive level” (P8, CN). Another therapist from Taiwan shared a similar observation that “They often said they don't have feelings or say they don't understand what I mean about feelings; they tend to say what they think or what actions they took” (P6, TW).

When situations like this arise, therapist shared that instead of continuing to ask partners to express and share emotions, they prioritize validating partners' cognition and understand why cognition is more accessible than emotions to foster emotion access and engagement. One therapist from mainland China shared: “They need to be understood for why they are so rational; it's due to cultural expectations” (P8, CN). Another therapist also emphasized the importance of adjusting therapeutic skills to facilitate emotional engagement:

It's not that men don't talk about emotions; it's that they need to be understood. They also need to understand why they are so rational—it's actually due to societal expectations. Once they begin to understand their rationality, and when they receive validation and understanding from therapists, they can start to talk about and share their emotions.

(P14, CN)

##### 6.2.2.2 Unpack Multilevel Emotions Gradually and Articulate Emotions for Couples

Therapists observed that one of the main reasons couples find it difficult to focus on emotions is due to unfamiliar experiences; for instance, they were never taught how to talk about emotions. Some individuals are willing to share their emotions but lack the vocabulary to articulate them. A therapist from mainland China observed:

They are really stuck… it's not that they don't want to respond or share emotions. It's not that they are indifferent or don't care—they just don't have the emotion vocabulary to share. Sometimes, when they share that they don't feel comfortable, it's already a big step for them in expressing their emotions.

(P18, CN)

And for some couples, even after they are finally able to share their emotions, they still struggle to respond to their partners' emotions, as a therapist in Taiwan shared: “Sharing emotions is one thing, and responding to their partner's emotions is another. This is especially prevalent in Chinese culture because they are not familiar with sharing and responding to emotions” (P20, HK).

In response to the lack of emotion vocabulary, many therapists shared that they had to go very slow to help clients unpack their emotions gradually. For example, a therapist shared that: “I often keep my emotion reflection [to client] subtle and slow, like Do you feel a bit like this sometimes? If he says yes, I ask him to say a bit more, slowly helping him to get used to expressing emotions by himself” (P10, CN).

Many therapists also shared that helping clients articulate their emotions to help them facilitate emotion access is helpful. Instead of asking clients to share and verbalize their emotions, therapists often need to offer an emotion word or an emotion description first, then check with clients if it resonates. A therapist shared:

My client shared a thought or an action, and my goal is to make them feel that coming to therapy is not so threatening so no matter what they said, I try my best to validate. Then, from their responses, I add some emotion or feeling‐related words, but not too much. If I use too many emotion words, they tend to deny it more easily…For example, if I respond by saying that he might feel conflicted between his wife and his mother‐in‐law, he might respond by saying that it's not really a big deal… I might say, “Okay, I understand that both of them are very important to you, and you don't often think about how you feel, but you try to make sure both of them are satisfied, and you feel that's good enough.” Then, he might agree and say, “Yes, that's exactly it.” From there, I slowly try to push forward and see if I can go a bit deeper of his emotions.

(P6, TW)

#### 6.2.3 Understanding Cultural Impacts on Couples' Presenting Problems

One of the main challenges therapists mentioned in interviews was that they often encountered scenarios specific to cultural expectations and values that were not addressed in EFCT books or materials. Overall, therapists believe that EFCT is still a helpful intervention for Chinese couples, if therapists are aware of the cultural impact on presenting problems and adjust the delivery of EFCT to better serve the needs and concerns of Chinese couples.

##### 6.2.3.1 Tolerance, Sacrifice, and Harmony Are Keys to Maintaining Relationships

Tolerance, sacrifice, and harmony were frequently mentioned by therapists as key characteristics of Chinese relationships. In Chinese culture, separation and divorce are still considered sources of shame or a loss of face for families. As a result, tolerance in relationships is often viewed as an appropriate strategy to maintain the relationship, even if it comes at the expense of one's own well‐being. Some couples even remind themselves of their partner's positive contributions to outweigh the difficulties of tolerance. One therapist shared a couple's account in therapy: “I can't tell people that my husband had an affair or express my frustration with him, no matter how hurtful it is, I have to tolerate…I need to remember his contribution to our family…Sharing bad things about him will bring a sense of shame to our family” (P24, HK). Another therapist also shared the nuances of tolerance in Chinese relationships:

Under the expectations of Chinese culture, it often seems that if you encounter problems in your marriage, you should find a way to solve them, tolerate them, and work things out together as a couple. Why does it have to end in divorce? Many people believe that everyone gets through challenges in marriages by tolerance, so why can't you? For many older generations, divorce is seen as both shameful and a financial loss, as divorce often involves dividing assets…divorce is often thought as something disastrous.

(P14, CN)

Because of this expectation of tolerance and maintaining harmony in relationships, this expectation is often mirrored in the therapy room. Couples aim to preserve harmony in the therapy environment and are reluctant to discuss difficult topics that might disrupt the equilibrium. A therapist noted: “But sometimes I feel it's quite challenging for me. For example, sometimes I evoke the pain and one partner gets very upset…[I have a couple and the husband] said ‘My wife was doing fine until you talked about [the negative experiences]—why are you bringing this up?’… There's a lot of pressure on me as a therapist” (P11, CN).

A therapist shared that an important skill therapists need to implement and adjust in therapy is demonstrating that couples can achieve a harmonious relationship by coming to therapy. This is something therapists working with Chinese couples should keep in mind, as couples ultimately seek harmony:

Chinese couples don't like conflict; they prefer a culture of harmony. They need to see proof that coming to the therapy room results in harmony. [They want to know that] it's okay to have conflict, but will it end in disharmony? That's the key [to successful treatment outcomes]. This is a critical aspect therapists must provide to gain trust from couples.

(P2, TW)

##### 6.2.3.2 Navigating Marital and In‐Law Expectations Is Challenging

Many therapists have shared that couples in therapy often struggle to balance their roles within the marriage and their responsibilities to their families of origin. For men, particularly if they are the eldest son or grandson, there is often an expectation to take care of their siblings and the entire family. For women, there is an expectation to demonstrate filial piety toward their mother‐ and father‐in‐law, even when this conflicts with their own needs and desires. A therapist emphasized that: “In conflicts between a wife and mother‐in‐law, (theoretically) the husband should stand up for his wife and confront his family of origin…I don't think it necessarily applies in our culture” (P4, TW). A therapist in Taiwan shared an insightful observation of the internal struggles faced by daughters‐in‐law:

I've encountered situations where the husband genuinely supports his wife and tells her, “It's okay, you don't need to go back to my parents' house for the New Year; I'll take the children back myself, and they'll be happier that way.” This creates a deeply conflicted state for the wife. On one hand, she wonders, “Really? Is this okay? Can I truly not go back to your parents' house?” On the other hand, she worries that by not going, she might seem like a bad daughter‐in‐law…The husband has to find a balance between being a husband and being a son, and this can be incredibly difficult for him. I feel this kind of situation would not be discussed in Sue Johnson's books because these issues don't exist in the same way in the West where they tend to advocate for clear boundaries with the family of origin.

(P3, TW)

When the dilemma between role expectations is discussed in therapy, therapists mentioned a few recommendations to better attend to the needs of Chinese couples. One therapist discussed the importance of understanding the impact of collectivism on Chinese couples: “EFT is more focused on individualism, it leans towards the Western culture of individualism…But in Chinese culture, it's impossible to avoid the concept of collectivism” (P7, TW).” Another therapist also shared that validating the partner's sense of filial piety is one of the important skills for couples to address the dilemma of filial piety. Recognizing and validating this important cultural value, rather than focusing on how it creates conflict, opens a door for meaningful discussion and help couples feel heard and understood.

##### 6.2.3.3 Traditional Gender Roles Still Prevail in Some Regions

Therapists noted that geographical differences influence the nature of presenting problems, particularly regarding gender role expectations. In some rural areas of mainland China, therapists still observe traditional gender roles shaping couples' relationships. These roles further compound the complexity of issues related to in‐law expectations, parenting, and intimacy, as a therapist shared: “[Some clients] Growing up in a small, economically disadvantaged village where traditional values are deeply rooted, these beliefs remain strong and hard to change” (P12, CN).

These traditional gender roles are very hard to change in therapy because they have contributed to the clients' sense of success, and they often do not feel a need to change. A therapist from mainland China shared.

Especially men growing up in this region, currently in a high‐ranking government position, there's a certain pride in the way he speaks and presents himself…no discussion on emotions and his traditional family values helped him reach his current status in life.

(P12, CN)

#### 6.2.4 Recommended Training Additions for Therapists Working With Chinese Couples

When therapists were asked if they wished to receive additional training beyond their EFCT training, they highlighted a few areas that could help address the specific needs of Chinese couples. Many therapists shared that since discussing emotions is neither taught nor familiar for many couples, it might be a good strategy to spend the first one or two sessions providing psychoeducation about the importance of emotions and their impact on relationships before starting the EFCT treatment. Therapists noted that it would be beneficial to receive training on how to conduct psychoeducation on emotions with couples. Other therapists also shared that a psychoeducation workshop on healthy relationships before EFCT treatment might be helpful. One therapist noted:

Sometimes [couples] get into relationships because most of their friends are in relationships, so they want one too, but they never think about what a relationship means to them or what they need from the relationship…because they have never had a good role model from their family of origin.

(P14, CN)

Another therapist emphasized the importance of teaching couples how family of origins or in‐laws expectations could impact their relationships, and that one aspect of a healthy relationship is maintaining healthy boundaries with their families of origin: “They also need some psychoeducation on family values… like when they get married, they have to leave their parents emotionally… but sometimes they still want to depend on their parents, [which causes a negative impact on their relationship]” (P12, CN). A therapist suggested that in a psychoeducation workshop, the following topics could be covered:

We can teach couples how to fall in love and maintain a healthy relationship, how to evaluate their interactions in a relationship, and how to make adjustments to interactions when needed… [Sue Johnson] have the *Hold Me Tight* group, but sometimes when couples start to attend these workshops, it's too late, like when they already have children, and it's too late to address these important issues about relationships.

(P10, CN)

## 7 Discussions

Infidelity, in‐laws' involvement in couple's relationships, parenting, couples' high conflict, trauma, sex issues, and depression and anxiety are reported by therapists in Asia as common presenting problems for Chinese couples seeking therapy. These findings align with the current literature, which highlights the effectiveness of couple therapy in addressing infidelity (Baucom et al. 2006), posttraumatic stress (Monson et al. 2012), sexual difficulties and challenges (McCarthy and Thestrup 2008), relationship distress often linked to high conflict (Doss et al. 2022; Roddy et al. 2020), and depression (Wittenborn et al. 2019).

A unique presenting problem among Chinese couples is the influence of in‐law involvement in their relationships, a concern that is relatively rare as a primary presenting problem in the United States or European countries (Epstein et al. 2005). Parenting also emerges as a significant presenting issue in our current study. This finding is consistent with a cross‐cultural study by Su et al. (2015), which found out that raising children was perceived as the most significant marital problem in Taiwan, whereas communication was the primary concern for couples in the United States. These differences suggest that while love and intimacy are prioritized in intimate relationships in Western countries, parent‐child relationships are prioritized in collective culture as a way to fulfill responsibility and obligation to family subsystems. This includes both relationships between adult children and their own parents, as well as between parents and their children (Lam et al. 2016). One observation noted by therapists in the interviews was that parenting disagreements among Chinese couples are often rooted in concerns about children's behavioral and academic performance. This aligns with the literature, which indicates that, in Chinese culture, couples often do not seek therapy for themselves but are more likely to pursue it for the benefit of their children (Sze et al. 2011).

Another key finding from our study is that therapists reported that EFCT helps partners with mental health symptoms—such as depression and anxiety—experience a reduction in symptoms. This progress stems from the process of the affected partner feeling supported and understood by their caring partner in the therapy room. At the same time, the caring partner gains a deeper understanding of how mental health challenges impact their relationship, often leading to a grieving phase as they come to terms with the reality that their relationship may not align with their original expectations. This dual process underscores EFCT's core goal: accessing and reprocessing underlying emotions to create new, meaningful interactions, positioning the relationship as a secure base for safety and comfort (Johnson 2019).

Therapists discussed the challenges of encouraging couples, particularly men, to access and engage with emotions. These challenges stem primarily from cultural and societal expectations that discourage men from sharing vulnerable emotions. Instead of persistently pushing couples to “actively engage with an emotional reality” (Johnson 2019, p. 37) and embrace emotional vulnerability—central to the success of EFCT, therapists adapted their skills to make this process less threatening. These adaptations included validating clients' preferences for cognitive processing, unpacking emotions slowly, and, at times, explicitly using empathic conjecture to name emotions for couples. Such strategies demonstrate a sensitivity to the cultural expectations of Chinese couples, for whom emotion suppression is often viewed as a positive relational strategy. In collectivistic cultures, emotion suppression is seen as a means of regulating emotions effectively and is even associated with better mental health outcomes, unlike in individualistic cultures (Kraus et al. 2024; Tamir et al. 2024). Notably, an EFCT outcome study with couples in Taiwan found that both partners' high emotional expressivity at intake is associated with decreases in relationship distress and depressive symptoms over time (Tseng et al. 2024). This indicates that, despite cultural norms support emotion suppression, emotion expression and engagement remain key to success of EFCT among Chinese couples. This highlights the importance for therapists to honor cultural influences on emotional expression and respect couples' pace in processing emotions, while still maintaining the core components of EFCT.

Another cultural expectation that emerged in couples' relationships is the emphasis on tolerance, sacrifice, and harmony as strategies for maintaining relational stability. Navigating boundaries with in‐laws also surfaced as a culturally specific issue frequently discussed in sessions. These dynamics are deeply rooted in collectivist values, where the needs and feelings of others are more important than one's own (Tseng and Li 2023). For example, in‐laws' involvement in a couple's decision‐making process is often considered normal, particularly when children are involved. Grandparents may view it as their responsibility to ensure the well‐being of their grandchildren, even if this means interfering with the couple's parenting (Hu and Peng 2015; Lam et al. 2016). Without an awareness of these cultural nuances, therapists risk misinterpreting these dynamics through a Western lens. In Western contexts, advocating for one's needs or setting boundaries with family members is often encouraged, as suppressing one's needs or avoiding conflict is seen as unhealthy. However, in Chinese cultural contexts, such actions may conflict with the values of family harmony and interconnectedness (Tseng and Li 2023). This highlights the importance of adapting EFCT skills that respect these values while supporting couples in finding balance within their relationships.

## 8 Limitation

One major limitation of this study is that, although we attempted to include therapists from different regions to capture a range of perspectives on Chinese couples receiving EFCT, there may still be heterogeneity within each region. Furthermore, while all participating therapists in this study identify as Chinese, their insights cannot fully represent the experiences of all Chinese couples, who themselves are highly diverse and heterogeneous.

## 9 Conclusion

This is the first qualitative study to explore therapists' perspectives on using EFCT with Chinese couples, focusing on their experiences regarding the fit and misfit of EFCT for this population. Our findings suggest that EFCT generally aligns well with Chinese couples, highlighting the importance of therapists understanding cultural values and nuances that influence presenting problems. Therapists should also adjust their EFCT skills to honor and understand clients' cultural values.

Notably, this study is one of the rare efforts to include perspectives from therapists across Taiwan, Hong Kong, and mainland China to offer a unique and comprehensive understanding of EFCT within Chinese cultural contexts. By bridging insights across these regions, despite their political tensions and territorial disputes, the study highlights the importance of prioritizing cultural and clinical considerations over geopolitical boundaries in advancing therapy practices. Future research on culturally adapted EFCT should continue to incorporate therapists' perspectives to further refine and develop culturally responsive approaches for this population.

## Supporting information


**Supplemental Table 1:** Interview Guide for Therapists Working with Chinese Couples in Asia.
