# Supplementary material for: Emotion Access and Navigation in Chinese Couples: Insights and Adaptations From Emotionally Focused Couples Therapy Therapists
Source: J Marital Fam Ther. 2025 Nov 21;52(1):e70093. doi: 10.1111/jmft.70093 (PMC12636061; doi:10.1111/jmft.70093)
Supplement: Supplementary file 1 — Supplemental Table 1: Interview Guide for Therapists Working with Chinese Couples in Asia. [file JMFT-52-0-s001.docx]

**Supplemental Table 1**

*Interview Guide for Therapists Working with Chinese Couples in Asia*

| **Question Number** | **Interview Question** |
| --- | --- |
| 1 | What does couple therapy look like in your region? |
| 1a | What are the common settings where couple therapy is typically conducted (e.g., private practice, agency, hospital)? |
| 1b | What are the typical fee structures for couple therapy sessions in your region, and how do they impact access to therapy for Chinese couples? |
| 1c | In your experience, what is the average length of treatment for Chinese couples? |
| 1d | What are some challenges you have observed in providing couple therapy in your region? |
| 2 | What are the common presenting problems you have encountered when working with Chinese couples in Asia? |
| 3 | In your clinical experience, has EFCT been helpful in improving clinical outcomes (e.g., depression, anxiety, etc.)? What other areas of improvement have you observed? |
| 4 | Research has shown the effectiveness of EFCT across different cultures. In your clinical experience, how well does EFCT fit Chinese culture? |
| 5 | What does “sharing vulnerability” look like when working with Chinese couples? |
| 6 | How can we improve or adapt EFCT to better address the needs of Chinese couples and families? |

**﻿**
